# Supplementary material for: When Reality Does Not Meet Expectations—Experiences and Perceived Attitudes of Dutch Stakeholders Regarding Payment and Reimbursement Models for High-Priced Hospital Drugs
Source: Int J Environ Res Public Health. 2022 Dec 26;20(1):340. doi: 10.3390/ijerph20010340 (PMC9819658; doi:10.3390/ijerph20010340)
Supplement: Supplementary file 1 [file ijerph-20-00340-s001.zip › ijerph-2064938-supplementary.pdf]

Supplementary Information

Table S1 – Perspective categorization of the different stakeholder groups

Table 1. Perspective categorization of the different stakeholder groups

Perspective
Ministerial
Ministry of Health
Health Technology Assessment agency
National Health Care Institute (ZIN)
Healthcare payers
Healthcare insurers
Pooled procurement
Healthcare provider
Private/academic hospitals
Pharmaceutical industry
Healthcare consultant
Academic
Interest groups
Medical specialist societies
Patient organization

Box S1– Definition of the included payment and reimbursement models

Box 1. Included reimbursement and payment models

**Financial-based reimbursement models**	
Discounts / rebates	Simple price discounts, publicly or confidentially agreed upon between the payer and manufacturer.
Budget threshold / dedicated funds	Maximum amount of reimbursement for an individual innovative treatment (budget threshold) or therapeutic area (dedicated funds) to cap total expenditures. Translates into maximum number of patients treated per year (utilization capping) or sharing of costs with the manufacturer or patients after pre-defined budget threshold has been exceeded.
Price-volume agreements	Drug prices are progressively lowered as more patients receive the treatment.
**Outcome-based reimbursement models**	
Value-based pricing	Setting the price of a new medicine and/or decide on reimbursement based on the therapeutic value that a therapy offers, usually assessed through health technology assessment (HTA). To compare value across healthcare domains incremental cost-effectiveness ratios and willingness to pay-thresholds can be used.
Pay-for outcome / outcome guarantees	The level of reimbursement is related to the future performance of the product in either a research or a real world (performance-based) environment. Therapy costs are fully or partially covered by the manufacturer if outcomes are not achieved.
Conditional treatment continuation	Continuation of coverage for individual patients is conditioned upon meeting short-term treatment goals. When agreed conditions are not met, coverage will end.
Coverage with evidence development	Provisional reimbursement of promising technologies with limited clinical evidence. Temporary reimbursement is granted with an obligation for the manufacturer to obtain and provide additional data. Can be organized either with patients only having access when included in the study (only in research) or with an obligation to generate data and unrestricted access (only with research).
**Upfront payment**	
	Agreement to pay treatment costs upfront to the manufacturer at time of treatment delivery. This is the most common payment model.
**Delayed payment models**	
Payments at outcome achieved	Paying treatment costs only after pre-defined results have been achieved.
Annuity Payments	Spreading payments over multiple years, with an agreement upon amount of treatment or outcomes delivered.
Health leasing / subscription	Paying for unlimited use of a therapy during a predefined period.

Supplementary Text1 – The questionnaire

Questionnaire. Payment and reimbursement models for high-priced hospital drugs in the Netherlands

Enquête naar vergoedingsovereenkomsten en betalingsregelingen voor dure monopolistische intramurale geneesmiddelen

Hartelijk dank dat u de tijd wil nemen om deze vragenlijst te beantwoorden.

Door middel van deze enquête willen wij graag onderzoeken hoe vergoedingsovereenkomsten en betalingsregelingen voor monopolistische dure intramurale geneesmiddelen (geneesmiddelen die in het ziekenhuis worden gebruikt) in Nederland door veldpartijen worden ervaren.

De focus
De focus van deze enquête ligt op ervaringen van veldpartijen die zowel direct en/of indirect betrokken zijn bij het maken van vergoedingsovereenkomsten en betalingsregelingen. Op deze manier willen wij een breed, maar ook diepgaand inzicht krijgen in de ervaringen van diverse veldpartijen en in waar zij sterke punten en barrières ervaren. Deze informatie kan gebruikt worden om de toekomstige implementatie van vergoedingsovereenkomsten en betalingsregelingen te verbeteren.

Deze vragenlijst is ontwikkeld door de Universiteit Utrecht als onderdeel van een project dat deel uitmaakt van de Academische Werkplaats ‘Verzekerde Zorg’ waar Zorginstituut Nederland (ZIN) de Universiteit Utrecht en de Erasmus Universiteit Rotterdam in samenwerken. De werkplaats heeft als doel een brug te vormen tussen onderzoek en beleid.

Kennisclip en begrippenlijst
Omdat de gebruikte begrippen voor vergoedingsovereenkomsten in de praktijk vaak verschillend worden gebruikt en geïnterpreteerd, zult u aan het begin van de enquête een filmpje te zien krijgen waarin wordt toegelicht hoe de begrippen in deze enquête zijn toegepast. De begrippen zijn in de survey ook altijd terug te vinden door rechts bovenin op het knopje 'vraagindex' te drukken en dan de begrippenlijst te openen. De begrippenlijst is tevens **hier** te downloaden.

Geschatte duur van de enquête

De vragenlijst is getest door vertegenwoordigers vanuit de Universiteit Utrecht en de Academische Werkplaats ‘Verzekerde Zorg’. Naar schatting zult u ongeveer 20 minuten nodig hebben om deze in te vullen.

Vragen of opmerkingen
Voor vragen of opmerkingen kun u contact opnemen met Marcelien Callenbach van de Universiteit Utrecht door te mailen naar M.H.E.Callenbach@uu.nl.

Let op: Open deze enquête in Firefox of in de Google Chrome. Als de online tool desondanks niet werkt, kunt u het Word-document gebruiken dat bij de e-mail is toegevoegd. Onder aan de pagina vindt u informatie over de gegevensbescherming. Door deze te accepteren kunt u naar de volgende pagina

Nogmaals hartelijk dank dat u de tijd neemt om deze vragen in te vullen.
Met vriendelijke groeten,
Namens de samenwerkende partners, Universiteit Utrecht (UU) en Zorginstituut Nederland (ZIN)

Marcelien Callenbach, MSc (PhD student),
Dr. Rick Vreman,
Prof. Dr. Aukje Mantel-Teeuwisse,
Dr. Wim Goettsch

Gegevensbescherming

Houd er rekening mee dat we deze vragenlijst niet volledig konden anonimiseren vanwege organisatorische en analytische doeleinden.
We willen u verzekeren dat we zorgvuldig met uw informatie omgaan en geen gegevens publiceren of delen met anderen die naar u persoonlijk of uw functie herleidbaar zijn, in overeenstemming met Europese wetgeving (EU) 2016/679. Door ons onderstaande beleid inzake enquêtegegevens te accepteren gaat u akkoord met deze voorwaarden.

○ **Ik ga akkoord met het beleid rondom de gegevensbescherming**

Kennisclip

Klik hier om de kennisclip te bekijken (kijktijd 6 minuten).

Begrippenlijst

Utrecht University

Pharmacoepidemiology
and Clinical Pharmacology

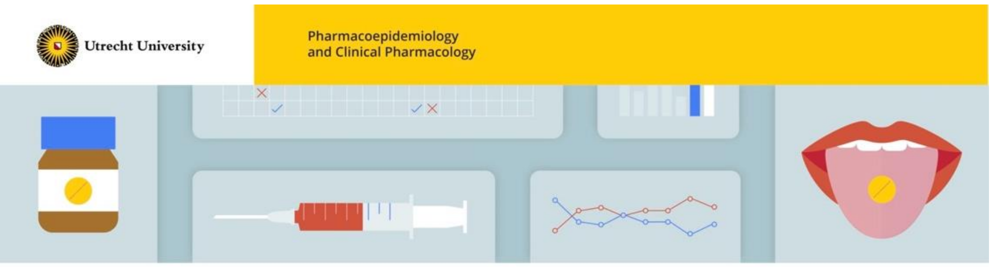

Definities
Vergoedingsovereenkomsten en betalingsregelingen

**Prijs-volume afspraak**	Prijs-volume afspraken zijn overeenkomsten waarbij prijzen gelinkt worden aan volume. Dit kan bijvoorbeeld inhouden dat de medicijn prijzen geleidelijk worden verlaagd naarmate meer patiënten de behandeling krijgen.
**Budget drempel**	Een budget drempel houdt in dat er een maximumbedrag is voor een individuele innovatieve behandeling (budgetdrempel) of dat er een maximumbedrag is therapeutisch gebied (speciale fondsen).
**Kortingen**	Dit zijn eenvoudige prijskortingen. Dit kan bijvoorbeeld in de vorm van een korting vooraf of een korting achteraf die door het bedrijf wordt terugbetaald.
**Waarde afhankelijke prijzen**	De prijs en/of vergoeding van een nieuw geneesmiddel wordt vastgesteld op basis van de (maatschappelijke) therapeutische waarde die het geneesmiddel biedt. Dit wordt doorgaans beoordeeld via health technology assessment (HTA). Om de waarde tussen zorgdomeinen te vergelijken kunnen incrementele kosteneffectiviteitsratio's en betalingsbereidheid gebruikt worden maar ook impliciete factoren als de zeldzaamheid van de ziekte.
**Voorwaardelijke vergoeding met bewijsvergaring**	Een overeenkomst voor tijdelijke vergoeding van veelbelovende technologieën met beperkt klinisch bewijs met de verplichting voor de fabrikant om aanvullende klinisch bewijs te genereren en te verschaffen. Op basis van deze aanvullende informatie kan zowel de vergoeding als de prijzen worden aangepast
**Betalen voor uitkomsten**	Een overeenkomst waarbij prijsniveau en/of de ontvangen inkomsten zijn gerelateerd aan de toekomstige prestaties van het geneesmiddel. De geneesmiddelkosten worden door de fabrikant verlaagd of kwijtgescholden als de resultaten niet worden bereikt.
**Voorwaardelijke voortzetting van behandelingen**	Een overeenkomst waarbij waarbij de voortzetting van de vergoeding voor individuele patiënten afhankelijk is van het behalen van bepaalde behandelingsdoelen op korte termijn. Wanneer geen effect van de behandeling bij de patiënt wordt aangetoond wordt de behandeling gestopt en zal er geen vergoeding plaatsvinden.

**Vooruitbetaling**	Een betalingsregeling waarbij op het moment van aflevering van het geneesmiddelen een betalingsverplichting aangegaan wordt.
**Betaling bij behaald resultaat**	Een betalingsregeling die inhoudt dat de behandelingskosten pas worden betaald nadat bepaalde, eerder afgesproken, resultaten zijn behaald.
**Gespreide betaling**	Dit is een regeling waar de betaling over meerdere jaren wordt verspreid in combinatie met een overeenkomst over de hoeveelheid geleverde behandelingen of resultaten.
**Abonnement**	Een betalingsregeling waar ongelimiteerd gebruik van een medicijn gemaakt kan worden tegen een vast bedrag per tijdsinterval. Dit bedrag is niet gekoppeld aan de waarde van de therapie, aangezien het gebruik onbeperkt is. Het tarief kan niettemin worden gebaseerd op een schatting van het gebruik en de verwachte voordelen.

Marcelien Callenbach Msc, prof. dr. Aukje Mantel-Teeuwisse Dr. Wim Goettsch en Dr. Rick Vreman

Promotieonderzoek: Vergoedings- en prijsarrangementen voor nieuwe dure geneesmiddelen met beperkt bewijs voor effectiviteit

Algemene vragen

De volgende vragen zijn bedoeld om algemene informatie te verzamelen over uw rol binnen het huidige proces van vergoedingsovereenkomsten voor dure monopolistische intramurale geneesmiddelen.

Vanuit het perspectief van welke organisatie/instelling waaraan u verbonden bent vult u deze vragenlijst in?
Kies één van de volgende mogelijkheden:

- Ministerie voor Volksgezondheid, Welzijn en Sport (VWS)
- Nederlandse Zorgautoriteit (NZa)
- Zorginstituut Nederland (ZIN)
- Overige overheidsinstelling
- Farmaceut
- Zorgverzekeraar
- Een gemeenschappelijk inkoopverband
- Medisch specialistische vereniging (inclusief beroeps- en wetenschappelijke vereniging en FMS)
- Ziekenhuis
- Patiëntorganisatie
- Consultancybureau
- Academie
- o Anders, namelijk _________________

Hoe zou u in het kort de voornaamste rol van uw organisatie binnen het proces van het maken van vergoedingsovereenkomsten en betalingsregelingen omschrijven? Mocht u of uw organisatie niet direct betrokken zijn bij het maken van deze overeenkomsten, beschrijf dan kort welke (indirecte) rol uw organisatie speelt.

……..………………..………………..…………………………………………………..

Welke doelstellingen worden volgens u, vanuit het perspectief van uw organisatie, het meest nagestreefd bij het tot stand komen van vergoedingsovereenkomsten voor dure monopolistische intramurale geneesmiddelen? (Meerdere antwoorden zijn mogelijk)

- Het verbeteren van toegang tot dure geneesmiddelen
- Het verlagen van de prijs waardoor de budget impact beperkt blijft of verlaagd wordt
- Het verlagen van de prijs waardoor de relatieve kosteneffectiviteit verbeterd wordt
- Het creëren van meer zekerheid in de relatieve kosteneffectiviteit door extra data te verzamelen
- Het adresseren van onvervulde medische behoefte
- Het bieden van een extra mogelijkheid aan fabrikanten om toegang te krijgen tot de markt
- Het onder controle houden van risico's en onzekerheden voor patiënten
- Het onder controle houden van financiële risico's en onzekerheden
- Het zorgen dat de behandeling gericht is op patiënten bij wie de kans het grootst is dat ze op een nieuw geneesmiddel zullen reageren
- Het delen van risico's met de betrokken partijen
- Het proces omtrent vergoedingsovereenkomsten en betalingsregelingen duidelijker, efficiënter en korter maken zodat er eerder en een meer duurzame toegang is tot geneesmiddelen
- Anders, namelijk __________________

Met welke soort organisaties werkt u/uw organisatie veel samen in het kader van vergoedingsovereenkomsten en betalingsregelingen voor dure monopolistische intramurale geneesmiddelen?

- Ministerie voor Volksgezondheid, Welzijn en Sport (VWS)
- Nederlandse Zorgautoriteit (NZa)
- Zorginstituut Nederland (ZIN)
- Overige overheidsinstelling
- Farmaceut
- Zorgverzekeraar
- Een gemeenschappelijk inkoopverband
- Medisch specialistische vereniging (inclusief beroeps- en wetenschappelijke vereniging en FMS)

- Ziekenhuis
- Patiëntorganisatie
- Consultancybureau
- Academie
- Anders, namelijk ___________________

Ervaringen met huidige vergoedingsovereenkomsten

De volgende vragen zijn bedoeld om uw ervaringen met de huidige vergoedingsovereenkomsten voor dure monopolistische intramurale geneesmiddelen te verkennen en inzicht te krijgen in welke sterke punten u hierbij ervaart.

Hoe vaak worden de onderstaande vergoedingsovereenkomsten voor dure monopolistische intramurale geneesmiddelen naar uw ervaring toegepast?
Kies het toepasselijke antwoord voor elk onderdeel:

	Nooit toegepast	Zelden toegepast	Soms toegepast	Vaak toegepast	Altijd toegepast	Weet ik niet
Prijs-volume afspraak						
Budgetdrempel						
Kortingen						
Waarde - afhankelijke prijzen						
Voorwaardelijke vergoeding met bewijsvergaring						
Betalen voor uitkomsten						

Voorwaardelijke voortzetting van behandelingen						

Met welke van de volgende vergoedingsovereenkomsten heeft u binnen het huidige Nederlandse systeem, de **beste** ervaringen?
Selecteer alle die van toepassing zijn en rankschik de geselecteerde opties. Kies maximaal 3 antwoorden.

**Vergoedingsovereenkomsten**	**Uw rangschikking**
• Prijs-volume afspraak<br>• Budgetdrempel<br>• Kortingen<br>• Waarde-afhankelijke prijzen<br>• Voorwaardelijke vergoeding met bewijsvergaring<br>• Betalen voor uitkomsten<br>• Voorwaardelijke voortzetting van behandelingen	1……<br>2……<br>3……

In de vorige vraag heeft u in de ranking één vergoedingsovereenkomst aangegeven waarmee u de beste ervaringen heeft. Welke van de volgende statements worden door u ervaren als voordelen van deze vergoedingsovereenkomst?
Als belangrijke voordelen van deze vergoedingsovereenkomst er niet bij staan, vult u dat dan alstublieft als vrije tekst in de onderstaande tekst vlak in. Selecteer alle die van toepassing zijn en rankschik de geselecteerde opties. Kies maximaal 5 antwoorden.

**Vergoedingsovereenkomsten**	**Uw rangschikking**
• De overeenkomst is eenvoudig te implementeren<br>• De overeenkomst is voor veel partijen acceptabel<br>• De overeenkomst brengt weinig administratieve lasten met zich mee<br>• De overeenkomst zorgt voor vroege toegang<br>• De overeenkomst is transparant	1……<br>2……<br>3……<br>4……<br>5…..

• De overeenkomst zorgt voor extra bewijsmateriaal<br>• De overeenkomst zorgt ervoor dat financiële risico's worden gedeeld<br>• De overeenkomst ondersteunt productprestaties door gericht gebruik te stimuleren<br>• De overeenkomst biedt de mogelijkheid om de prijsstelling van geneesmiddelen duidelijker te koppelen aan de prestaties<br>• De overeenkomst biedt de mogelijkheid een concurrentievoordeel te behalen<br>• De overeenkomst biedt de mogelijkheid om betere prijzen te krijgen dan de gepubliceerde prijzen van fabrikanten	

Verdere belangrijke voordelen:

…………………….………………………….…………………….………………
………

Ervaringen met huidige vergoedingsovereenkomsten

De volgende vragen zijn bedoeld om uw ervaringen met de huidige vergoedingsovereenkomsten voor dure monopolistische intramurale geneesmiddelen te verkennen en inzicht te krijgen in welke barrières u hierbij ervaart.

Rondom welke van de volgende vergoedingsovereenkomsten binnen het Nederlandse systeem ervaart u op dit moment barrières, welke u toch graag meer zou zien?

Meerdere antwoorden zijn mogelijk.

- o Prijs-volume afspraak
- o Budgetdrempel
- o Kortingen
- o Waarde-afhankelijke prijzen
- o Voorwaardelijke vergoeding met bewijsvergaring
- o Betalen voor uitkomsten
- o Voorwaardelijke voortzetting van behandelingen
- o Anders, namelijk __________________

Let op! Beantwoord bij de onderstaande vragen alleen de vragen met de vergoedingsovereenkomsten die u in de vorige vraag selecteerde.

Wat zijn de voornaamste barrières die u ervaart bij de vergoedingsovereenkomst vorm **prijs-volume afspraken**?
Meerdere antwoorden zijn mogelijk.

- o De overeenkomst komt lastig tot stand omdat er weinig samenwerking is tussen betrokken partijen.
- o De overeenkomst brengt hoge administratieve lasten met zich mee.
- o De regeling levert uiteindelijk niet het gewenste doel op.
- o De overeenkomst brengt tijdrovende onderhandelingen met zich mee.
- o Te weinig capaciteit beschikbaar om de overeenkomst op de gewenste manier te implementeren.
- o Het genereren van bewijs is in de praktijk lastig.
- o Gebrek aan transparantie.
- o Gegevensbeschermingskwesties.
- o Te grote financiële onvoorspelbaarheid.
- o Onzekerheid of de best mogelijke prijs wordt bereikt.
- o Verantwoordelijkheden zijn lastig te bepalen.
- o Anders, namelijk __________________

Wat zijn de voornaamste barrières die u ervaart bij de vergoedingsovereenkomst vorm **budgetdrempel**?
Meerdere antwoorden zijn mogelijk.

- o De overeenkomst komt lastig tot stand omdat er weinig samenwerking is tussen betrokken partijen.
- o De overeenkomst brengt hoge administratieve lasten met zich mee.

- o De regeling levert uiteindelijk niet het gewenste doel op.
- o De overeenkomst brengt tijdrovende onderhandelingen met zich mee.
- o Te weinig capaciteit beschikbaar om de overeenkomst op de gewenste manier te implementeren.
- o Het generen van bewijs is in de praktijk lastig.
- o Gebrek aan transparantie.
- o Gegevensbeschermingskwesties.
- o Te grote financiële onvoorspelbaarheid.
- o Onzekerheid of de best mogelijke prijs wordt bereikt.
- o Verantwoordelijkheden zijn lastig te bepalen.
- o Anders, namelijk ________________

Wat zijn de voornaamste barrières die u ervaart bij de vergoedingsovereenkomst vorm **kortingen**?
Meerdere antwoorden zijn mogelijk.

- o De overeenkomst komt lastig tot stand omdat er weinig samenwerking is tussen betrokken partijen.
- o De overeenkomst brengt hoge administratieve lasten met zich mee.
- o De regeling levert uiteindelijk niet het gewenste doel op.
- o De overeenkomst brengt tijdrovende onderhandelingen met zich mee.
- o Te weinig capaciteit beschikbaar om de overeenkomst op de gewenste manier te implementeren.
- o Het generen van bewijs is in de praktijk lastig.
- o Gebrek aan transparantie.
- o Gegevensbeschermingskwesties.
- o Te grote financiële onvoorspelbaarheid.
- o Onzekerheid of de best mogelijke prijs wordt bereikt.
- o Verantwoordelijkheden zijn lastig te bepalen.
- o Anders, namelijk ________________

Wat zijn de voornaamste barrières die u ervaart bij de vergoedingsovereenkomst vorm **waarde-afhankelijke prijzen**?

Meerdere antwoorden zijn mogelijk.

- o De overeenkomst komt lastig tot stand omdat er weinig samenwerking is tussen betrokken partijen.
- o De overeenkomst brengt hoge administratieve lasten met zich mee.
- o De regeling levert uiteindelijk niet het gewenste doel op.
- o De overeenkomst brengt tijdrovende onderhandelingen met zich mee.
- o Te weinig capaciteit beschikbaar om de overeenkomst op de gewenste manier te implementeren.
- o Het generen van bewijs is in de praktijk lastig.
- o Gebrek aan transparantie.
- o Gegevensbeschermingskwesties.
- o Te grote financiële onvoorspelbaarheid.
- o Onzekerheid of de best mogelijke prijs wordt bereikt.
- o Verantwoordelijkheden zijn lastig te bepalen.
- o Anders, namelijk __________________

Wat zijn de voornaamste barrières die u ervaart bij de vergoedingsovereenkomst vorm **voorwaardelijke vergoeding met bewijsvergaring**?
Meerdere antwoorden zijn mogelijk.

- o De overeenkomst komt lastig tot stand omdat er weinig samenwerking is tussen betrokken partijen.
- o De overeenkomst brengt hoge administratieve lasten met zich mee.
- o De regeling levert uiteindelijk niet het gewenste doel op.
- o De overeenkomst brengt tijdrovende onderhandelingen met zich mee.
- o Te weinig capaciteit beschikbaar om de overeenkomst op de gewenste manier te implementeren.
- o Het generen van bewijs is in de praktijk lastig.
- o Gebrek aan transparantie.
- o Gegevensbeschermingskwesties.
- o Te grote financiële onvoorspelbaarheid.
- o Onzekerheid of de best mogelijke prijs wordt bereikt.
- o Verantwoordelijkheden zijn lastig te bepalen.
- o Anders, namelijk __________________

Wat zijn de voornaamste barrières die u ervaart bij de vergoedingsovereenkomst vorm **betalen voor uitkomsten**?
Meerdere antwoorden zijn mogelijk.

- De overeenkomst komt lastig tot stand omdat er weinig samenwerking is tussen betrokken partijen.
- De overeenkomst brengt hoge administratieve lasten met zich mee.
- De regeling levert uiteindelijk niet het gewenste doel op.
- De overeenkomst brengt tijdrovende onderhandelingen met zich mee.
- Te weinig capaciteit beschikbaar om de overeenkomst op de gewenste manier te implementeren.
- Het generen van bewijs is in de praktijk lastig.
- Gebrek aan transparantie.
- Gegevensbeschermingskwesties.
- Te grote financiële onvoorspelbaarheid.
- Onzekerheid of de best mogelijke prijs wordt bereikt.
- Verantwoordelijkheden zijn lastig te bepalen.
- Anders, namelijk ___________________

Wat zijn de voornaamste barrières die u ervaart bij de vergoedingsovereenkomst vorm **voorwaardelijke voortzetting van behandeling**?
Meerdere antwoorden zijn mogelijk.

- De overeenkomst komt lastig tot stand omdat er weinig samenwerking is tussen betrokken partijen.
- De overeenkomst brengt hoge administratieve lasten met zich mee.
- De regeling levert uiteindelijk niet het gewenste doel op.
- De overeenkomst brengt tijdrovende onderhandelingen met zich mee.
- Te weinig capaciteit beschikbaar om de overeenkomst op de gewenste manier te implementeren.
- Het generen van bewijs is in de praktijk lastig.
- Gebrek aan transparantie.
- Gegevensbeschermingskwesties.
- Te grote financiële onvoorspelbaarheid.
- Onzekerheid of de best mogelijke prijs wordt bereikt.
- Verantwoordelijkheden zijn lastig te bepalen.
- Anders, namelijk ___________________

Wat is naar uw mening een goede oplossing of goed alternatief om deze barrière(s) in de toekomst weg te nemen?

……………………..…………………………..…………………………..……………………
……….

Bij welke soort producten ervaart u de grootste moeilijkheden om passende vergoedingsovereenkomsten te bepalen?
Kies niet meer dan 3 items.

**Soorten producten**	**Uw rangschikking**
• Een combinatie van therapieën<br>• Wanneer een therapie binnen veel verschillende mogelijke behandelsequenties kan worden toegepast<br>• Gepersonaliseerde geneesmiddelen, gebaseerd op een biomarker of gen<br>• Weesgeneesmiddelen<br>• Een preventieve behandeling of vaccin<br>• Histologie-onafhankelijke behandelingen<br>• Geneesmiddel voor een geavanceerde therapie (advanced therapy medicinal product, ATMP) zoals somatische celtherapie, genezende gentherapieën, weefselmanipulatie-geneesmiddelen	1……<br>2……<br>3……

Ervaringen met huidige betalingsregelingen

Vergoedingsovereenkomsten hebben vaak ook een betalingscomponent. Hierbij worden naast afspraken over de vergoeding ook afspraken gemaakt over bijvoorbeeld het moment van betalen en waaraan de betaling gekoppeld is. De volgende vragen zijn bedoeld om uw ervaringen met de huidige betalingsregelingen voor dure monopolistische intramurale geneesmiddelen te verkennen en inzicht te krijgen in welke sterke punten u hierbij ervaart.

Hoe vaak worden de onderstaande betalingsregelingen voor dure monopolistische intramurale geneesmiddelen naar uw ervaring toegepast?
Kies het toepasselijke antwoord voor elk onderdeel:

	Nooit toegepast	Zelden toegepast	Soms toegepast	Vaak toegepast	Altijd toegepast	Weet ik niet
Vooruitbetaling						
Betaling bij behaald resultaat						
Gespreide betaling						
Abonnement						

Met welke van de volgende vormen van betalingsregelingen heeft u, binnen het huidige Nederlandse systeem, de **beste** ervaringen?
Selecteer alle die van toepassing zijn en rank de geselecteerde opties. Kies niet meer dan 3 items.

**Betalingsregelingen**	**Uw rangschikking**
• Vooruitbetaling<br>• Betaling bij behaald resultaat<br>• Gespreide betaling<br>• Abonnement	1……<br>2……<br>3….

In de vorige vraag heeft u in de ranking aan gegeven met één betalingsregeling vorm de beste ervaringen te hebben. Welke van de volgende statements worden door u ervaren als voordelen van deze betalingsregeling?
Selecteer alle die van toepassing zijn en rank de geselecteerde opties.
Als belangrijke voordelen van deze betalingsregeling er niet bij staan, vult u dat dan alstublieft als vrije tekst in de onderstaande tekst vlak in.

**Betalingsregelingen**	**Uw rangschikking**
Makkelijk te implementeren<br>Goede spreiding van risico's<br>Acceptabel mechanisme voor de betrokken partijen<br>Makkelijk te combineren met andere vormen afspraken<br>Voorkomt hoge pieken in kosten<br>De regeling zorgt ervoor dat het risico met de fabrikant wordt gedeeld als het product niet werkt zoals afgesproken<br>Beperkt de totale impact op het budget	1……<br>2……<br>3……<br>4……<br>5…..

Overige belangrijke positieve punten:

…………………….……………………….…………………….……………
……….

Ervaringen met huidige betalingsregelingen

De volgende vragen zijn bedoeld om uw ervaringen met de huidige betalingsregelingen voor dure monopolistische intramurale geneesmiddelen te verkennen en inzicht te krijgen in welke barrières u hierbij ervaart.

Rondom welke van de volgende betalingsregelingen binnen het Nederlandse systeem ervaart u op dit moment barrières, welke u toch graag meer zou zien?
Meerdere antwoorden zijn mogelijk

- o Vooruitbetaling
- o Betaling bij behaald resultaat
- o Gespreide betaling
- o Abonnement
- o Anders, namelijk __________________

Let op! Beantwoord van de onderstaande vragen alleen de vragen met de vergoedingsovereenkomsten die u in de vorige vraag selecteerde.

Wat zijn de voornaamste barrières die u ervaart bij de betalingsregeling **vooruitbetaling?**
Meerdere antwoorden zijn mogelijk.

- o De regeling zorgt voor (incidentele) hoge financiële lasten
- o De regeling levert uiteindelijk niet het gewenste doel op
- o De regeling is moeilijk te combineren met andere afspraken
- o Te weinig capaciteit om de regeling op de gewenste manier te implementeren
- o Gebrek aan transparantie
- o De overeenkomst komt lastig tot stand omdat er weinig samenwerking is tussen betrokken partijen
- o De regeling is administratief moeilijk te regelen
- o Onduidelijkheden waar de initiële investering vandaan komt
- o Lastig om de precieze uitkomsten criteria te bepalen waaraan de betaling gerelateerd is
- o Anders, namelijk _________________

Wat zijn de voornaamste barrières die u ervaart bij de betalingsregeling **betaling bij behaald resultaat?**
Meerdere antwoorden zijn mogelijk.

- o De regeling zorgt voor (incidentele) hoge financiële lasten
- o De regeling levert uiteindelijk niet het gewenste doel op
- o De regeling is moeilijk te combineren met andere afspraken
- o Te weinig capaciteit om de regeling op de gewenste manier te implementeren
- o Gebrek aan transparantie
- o De overeenkomst komt lastig tot stand omdat er weinig samenwerking is tussen betrokken partijen
- o De regeling is administratief moeilijk te regelen

- Onduidelijkheden waar de initiële investering vandaan komt
- Lastig om de precieze uitkomsten criteria te bepalen waaraan de betaling gerelateerd is
- Anders, namelijk ________________

Wat zijn de voornaamste barrières die u ervaart bij de betalingsregeling **gespreide betaling?**
Meerdere antwoorden zijn mogelijk.
- De regeling zorgt voor (incidentele) hoge financiële lasten
- De regeling levert uiteindelijk niet het gewenste doel op
- De regeling is moeilijk te combineren met andere afspraken
- Te weinig capaciteit om de regeling op de gewenste manier te implementeren
- Gebrek aan transparantie
- De overeenkomst komt lastig tot stand omdat er weinig samenwerking is tussen betrokken partijen
- De regeling is administratief moeilijk te regelen
- Onduidelijkheden waar de initiële investering vandaan komt
- Lastig om de precieze uitkomsten criteria te bepalen waaraan de betaling gerelateerd is
- Anders, namelijk ________________

Wat zijn de voornaamste barrières die u ervaart bij de betalingsregeling **abonnement?**
Meerdere antwoorden zijn mogelijk.
- De regeling zorgt voor (incidentele) hoge financiële lasten
- De regeling levert uiteindelijk niet het gewenste doel op
- De regeling is moeilijk te combineren met andere afspraken
- Te weinig capaciteit om de regeling op de gewenste manier te implementeren
- Gebrek aan transparantie
- De overeenkomst komt lastig tot stand omdat er weinig samenwerking is tussen betrokken partijen
- De regeling is administratief moeilijk te regelen
- Onduidelijkheden waar de initiële investering vandaan komt
- Lastig om de precieze uitkomsten criteria te bepalen waaraan de betaling gerelateerd is
- Anders, namelijk ________________

Wat is naar uw mening een goede oplossing of alternatief om deze barrière(s) in de toekomst weg te nemen?

……………………….……………………………….…………………………….…………………
……….

Bij welke soort producten ervaart u de grootste moeilijkheden om passende betalingsregelingen te bepalen?
Kies niet meer dan 3 items.

**Soorten producten**	**Uw rangschikking**
• Een combinatie van therapieën<br>• Wanneer een therapie binnen veel verschillende mogelijke behandelsequenties kan worden toegepast<br>• Gepersonaliseerde geneesmiddelen, gebaseerd op een biomarker of gen<br>• Weesgeneesmiddelen<br>• Een preventieve behandeling of vaccin<br>• Histologie-onafhankelijke behandelingen<br>• Geneesmiddel voor een geavanceerde therapie (advanced therapy medicinal product, ATMP) zoals somatische celtherapie, genezende gentherapieën, weefselmanipulatie-geneesmiddelen	1……<br>2……<br>3……

Vergoedingsovereenkomsten en betalingsregelingen binnen de Nederlandse setting

De volgende vragen zijn bedoeld om inzicht te krijgen in uw mening ten aanzien van hoe vergoedingsovereenkomsten en betalingsregelingen in Nederland georganiseerd zijn. Hierbij richt de eerste vraag zich op het proces en de volgordelijkheid van het maken van vergoedingsovereenkomsten en betalingsregelingen. De daaropvolgende vragen focussen zich op de rol die de verschillende veldpartijen in het proces van vergoedingsovereenkomsten en betalingsregelingen spelen en hun onderlinge verhoudingen.

Bent u tevreden met hoe het maken van vergoedingsovereenkomsten en betalingsregelingen voor dure monopolistische intramurale geneesmiddelen in Nederland georganiseerd is?
Kunt u toelichten wat u goed of slecht vindt? (Denk bijvoorbeeld aan het proces binnen de sluisprocedure of juist voor de geneesmiddelen die daarbuiten vallen)

- o Zeer tevreden
- o Enigszins tevreden
- o Niet tevreden en niet ontevreden
- o Enigszins ontevreden
- o Zeer ontevreden

Geef hier een toelichting op uw antwoord:

……………………….……………………………….……………………….……………………….

Bent u tevreden met de rol van uw organisatie in het beleid rond vergoedingsovereenkomsten en betalingsregelingen in Nederland?
Kunt u toelichten wat u goed of slecht vindt?

- o Zeer tevreden
- o Enigszins tevreden
- o Niet tevreden en niet ontevreden
- o Enigszins ontevreden
- o Zeer ontevreden

Geef hier een toelichting op uw antwoord:

……………………….……………………………….……………………….……………………….

Hoe ervaart u de huidige manier van samenwerking tussen organisaties in het kader van vergoedingsovereenkomsten en betalingsregelingen? (Denk hierbij bijvoorbeeld aan het delen van informatie, transparantie en het gezamenlijk optrekken bij het maken of implementeren van vergoedingsovereenkomsten).
Kunt u toelichten wat u goed of slecht vindt?

- o Zeer tevreden

- Enigszins tevreden
- Niet tevreden en niet ontevreden
- Enigszins ontevreden
- Zeer ontevreden

Geef hier een toelichting op uw antwoord:

……………………….…………………………………….……………………….………………………

Op welk gebied, in het proces van vergoedingsovereenkomsten en betalingsregelingen, zit volgens u de meeste ruimte voor verbetering? Mocht u hier nog een antwoord aan toe willen voegen kan dat in de onderstaande tekstvlak.
Kies niet meer dan 5 items.

**Soorten producten**	**Uw rangschikking**
De manier waarop de afspraken tot stand komen<br>De implementatie van de afspraken<br>De evaluatie van afspraken<br>De manier waarop organisaties samenwerken<br>De mate van transparantie van gegevens<br>De mate van kennis en ervaring bij besluitnemers<br>De mate van kennis en ervaring bij de uitvoerders<br>De mate van inzicht in elkaars belangen<br>De ruimte in de huidige wetgeving<br>De focus op internationale samenwerking<br>Het vormen en afspreken van duidelijke randvoorwaarden<br>De manier waarop de afspraken tot stand komen<br>De implementatie van de afspraken<br>De evaluatie van afspraken<br>De manier waarop organisaties samenwerken<br>De mate van transparantie van gegevens<br>De mate van kennis en ervaring bij besluitnemers<br>De mate van kennis en ervaring bij de uitvoerders<br>De mate van inzicht in elkaars belangen<br>De ruimte in de huidige wetgeving<br>De focus op internationale samenwerking<br>Het vormen en afspreken van duidelijke randvoorwaarden	1……<br>2……<br>3……<br>4……<br>5……

Mocht u een antwoord toe willen voegen:

………………………..…………………………..………………………..…………
……….

Afsluiting

Deze enquête zal gevolgd worden door gesprekken. Mocht u hiervoor openstaan zou dat heel erg gewaardeerd worden. U kunt uw e-mailadres hieronder invullen.

………………………..…………………………..………………………..…………
……….

Mocht u suggesties voor organisaties of personen hebben die relevant zouden kunnen zijn om te benaderen om hun ervaringen met vergoedingsovereenkomsten en betalingsregelingen te delen, kunt u dat hieronder aangeven.

………………………..…………………………..………………………..…………
……….

Hartelijk dank voor het invullen van deze vragenlijst.

Voor vragen of opmerkingen kun u contact opnemen met Marcelien Callenbach van de Universiteit Utrecht door te mailen naar M.H.E.Callenbach@uu.nl.

Verzend uw enquête.
Bedankt voor uw deelname aan deze enquête.
